# Circadian rhythm-associated lncRNA RP11-414H17.5 as a key therapeutic target in osteosarcoma affects the tumor immune microenvironment and enhances malignancy

**DOI:** 10.1186/s13018-023-04442-9

**Published:** 2023-12-09

**Authors:** Liangkun Huang, Wanting Liang, Wenxiang Cai, Hao Peng

**Affiliations:** 1 Department of Orthopedics Surgery, Renmin Hospital of Wuhan University, Wuhan, 430060 Hubei China; 2 Department of Clinical Medicine, Xiamen Medical College, Xiamen, 310058 China

**Keywords:** Long non-coding RNA, Osteosarcoma, Metastasis, Prognosis, Circadian rhythm, Immune infiltration

## Abstract

**Background:**

It has previously been proven that circadian rhythm disruption is associated with the incidence and deterioration of several tumors, which potentially leads to increased tumor susceptibility and a worse prognosis for tumor-bearing patients. However, their potential role in osteosarcoma has yet to be sufficiently investigated.

**Methods:**

Transcriptomic and clinical data of 84 osteosarcoma samples and 70 normal bone tissue samples were obtained from the TARGET and GTEx databases, circadian rhythm-related genes were obtained from Genecards, and circadian rhythm-related lncRNAs (CRLs) were obtained by Pearson correlation analysis, differential expression analysis, and protein–protein interaction (PPI) analysis. COX regression and LASSO regression were performed on the CRLs in order to construct a circadian rhythm-related prognostic prediction signature (CRPS). CRPS reliability was verified by Kaplan–Meier (KM), principal component analysis (PCA), nomogram, and receiver operating characteristic (ROC) curve. CRPS effects on the immune microenvironment of osteosarcoma were explored by enrichment analysis and immune infiltration analysis, and the effect of critical gene RP11-414H17.5 on osteosarcoma was experimentally verified.

**Result:**

CRPS consisting of three CRLs was constructed and its area under the curve (AUC) values predicted that osteosarcoma prognosis reached 0.892 in the training group and 0.843 in the test group, with a p value of < 0.05 for the KM curve and stable performance across different clinical subgroups. PCA analysis found that CRPS could significantly distinguish between different risk subgroups, and exhibited excellent performance in the prediction of the immune microenvironment. The experiment verified that RP11-414H17.5 can promote metastasis and inhibit apoptosis of osteosarcoma cells.

**Conclusion:**

The study revealed that circadian rhythm plays a crucial role in osteosarcoma progression and identified the impact of the key gene RP11-414H17.5 on osteosarcoma, which provides novel insights into osteosarcoma diagnosis and therapy.

## Introduction

Osteosarcoma is a rare malignant tumor of bone that typically occurs in people between the ages of 10 and 20 [1]. The current treatment for osteosarcoma is a combination of surgical resection and chemotherapy, and both pre- and postoperative chemotherapy is required, but for osteosarcoma patients who develop distant metastases, these means are not effective for improving the prognosis which is extremely unsatisfactory [2–4]. Therefore, identifying new therapeutic targets and methods for the prediction of osteosarcoma metastasis is an important research topic. Long non-coding RNA (lncRNA) is an RNA over 200 nucleotides in length that does not encode proteins and engages in a variety of biological processes that include cell development and differentiation, chromatin modification, and transcriptional activation and regulation [5–7]. An increasing number of studies have recently confirmed that lncRNAs play an essential part in tumor progression and may serve as diagnostic and therapeutic targets for different tumors [8–11]. lncRNAs can affect cancer progression by regulating protein activity, transcriptional interference, and mediating histone modifications [12, 13]. Zhu et al. [15] discovered an effect of lncRNA SNHG10 on osteosarcoma invasion and proliferation through the regulation of FZD3. The rhythmic nature of physiological and physiochemical features in organisms is regulated by circadian rhythm, which has also been confirmed to affect immune parameters including macrophage and T cell counts, which regulate the homeostasis of the immune system [16–20]. A disturbed circadian rhythm can also affect the biological properties of tumor cells, which can lead to the deterioration of different tumors [21, 22], and affect biological processes including cell proliferation, apoptosis, cell cycle, and DNA damage response [23, 24]. Circadian rhythm disruption has also been confirmed to play a crucial role in the progression of glioblastoma [25], lung cancer [26], and breast cancer [27]. Therefore, circadian rhythm-related gene regulation may be a new diagnostic strategy for osteosarcoma, but there is no published research related to the role of circadian rhythms in osteosarcoma. The aim of this study is to investigate the prognostic value of differentially expressed circadian rhythm-associated lncRNAs in osteosarcoma, as well as their guiding significance in the immune microenvironment and drug sensitivity. We successfully constructed an osteosarcoma prognostic prediction model consisting of three lncRNAs, which performed stably in predicting osteosarcoma prognosis and metastasis. The function of RP11-414H17.5 in metastasis of osteosarcoma promotion was discovered and well validated experimentally. In addition, the correlations among the tumor immunological microenvironment, prognostic model, clinical features, and drug sensitivity were probed.

## Materials and methods

### Obtaining the necessary data

We obtained the research trends of circadian rhythm-related pathways in recent years by R package enrichplot. A total of 943 circadian rhythm-related genes were acquired from the Genecards website (https://www.genecards.org/, set to correlation score > 1) and osteosarcoma and normal bone tissue samples were obtained from the TARGET and GTEx databases (https://xena.ucsc.edu/). The required data included transcriptomic data, survival data, tumor metastasis, age, and sex. A total of 84 osteosarcoma samples and 70 normal bone tissue samples fulfilled the requirements. All osteosarcoma samples were randomly divided into training and test sets at 3:2 ratio.

### Screening differentially expressed lncRNAs associated with circadian rhythm

Differential expression analysis was performed on osteosarcoma and normal bone tissue samples using the R package DESeq2 (log2foldchange > 0.8, adjusted p value < 0.05) as a means of obtaining differentially expressed genes (DEGs). The 943 circadian rhythm-related genes were subjected to PPI and Cytoscape joint analysis (set minimum interactive score of 0.7), and genes with betweenness\closeness\degree values in the top 50% were taken and intersected with DEGs to acquire differentially expressed circadian rhythm hub genes (DEcir hub genes). Pearson correlation analysis identified correlations between hub genes and lncRNAs (R > 0.4, p < 0.001) and they were then intersected with DEGs in order to obtain differentially expressed circadian rhythm-associated lncRNAs (DECRLs).

### Constructing a prognostic predictive risk model CRPS

The clinical data and transcriptomic data of 51 TARGET samples (the training set) were combined to perform univariate COX regression assays using R packages survminer and survival. Screening criterion was set at p < 0.05. LASSO regression analyses were then performed on the results using the R package glmnet with 1,000 cross-validations to obtain six candidate DECRLs. Finally, multivariate COX regression assay was performed and the three DECRLs with the highest prognostic predictive value were generated, which led to the construction of a risk model, CRPS. The risk scores of all osteosarcoma samples were determined by the following formula: ${\varvec{R}}{\varvec{i}}{\varvec{s}}{\varvec{k}}{\varvec{S}}{\varvec{c}}{\varvec{o}}{\varvec{r}}{\varvec{e}}={\sum }_{{\varvec{i}}=1}^{{\varvec{n}}}{{{\varvec{C}}{\varvec{o}}{\varvec{e}}{\varvec{f}}}_{{\varvec{i}}}^{*}{\varvec{X}}}_{{\varvec{i}}}$. Coef denotes the risk coefficient value and Xi denotes the standardized expression of the three DECRLs. All osteosarcoma samples were classified into different risk subgroups based on risk score median value. The different risk subgroups obtained were used for subsequent analyses.

### Assessing CRPS prognostic predictive power

Survival state curves and scatter plots were plotted in the expression heat maps of the three DECRLs for the training group samples. Using the R package ROCR, the ROC curves were plotted at 1, 3, and 5 years and the AUC value was calculated to determine CRPS reliability for forecasting the prognosis of patients. ROC curves of different clinical characteristics were then plotted to compare with them. The KM curve was plotted as a means of identifying whether the survival of different risk subgroups is statistically different using the R package survminer (p < 0.05). Finally, univariate and multivariate COX regression analyses were performed on CRPS as well as different clinical characteristics for the assessment of the impact of these variables on patient subsistence using R package survival. PCA was conducted to verify the discriminatory ability of CRPS. The same analysis was performed on test sets and all cohorts in order to verify the stability of CRPS prediction capability.

### Assessing CRPS performance in different clinical subgroups

In order to investigate whether CRPS has a stable performance in patients with different clinical characteristics, osteosarcoma patients were grouped according to their clinical characteristics (age, gender, whether they were metastatic or not), and then, KM curves were plotted for analyzing whether different clinical characteristics affected the applicability of this prognostic prediction model.

### Constructing a nomogram

A nomogram was constructed based on clinical characteristics (age, gender, whether or not they were metastatic) and CRPS to assess 1-, 3-, and 5-year survival rates in osteosarcoma samples using R package rms. Calibration curves were then plotted to determine the predictive reliability of this nomogram. Finally, the predictive power of this nomogram and other metrics for patient survival was determined using C-index analysis and ROC curves.

### Examining the correlation between DECRLs and osteosarcoma metastasis

Box plots were drawn using R package ggstatsplot for investigating the correlation of DECRLs with metastasis. ROC curves and scatter plots of the correlation between DECRLs and osteosarcoma metastasis-related genes were plotted to validate the association of DECRLs with osteosarcoma metastasis.

### Performing enrichment analysis on genes

Differential expression analysis was conducted for different risk subgroups. The DEGs that were obtained were analyzed using Gene Ontology (GO) and Kyoto Encyclopedia of Genes and Genomes (KEGG) enrichment with R package clusterProfiler (the screening criterion: adjusted p value < 0.05) as a means of investigating the biological pathways that may be involved.

### Performing immune microenvironment and immune checkpoint analysis

Immune infiltration analysis and immune function scoring of osteosarcoma samples were performed to probe the association of CRPS with immune checkpoints and immune infiltration using R package GSVA, which assessed the differences of tumor immune microenvironment in different risk subgroups. R package limma was then used for conducting immune checkpoint correlation analyses.

### Sensitivity analysis of chemotherapeutic agents

The sensitivity of chemotherapeutic agents is expressed through half maximal inhibitory concentration (IC50) value [28]. IC50 is an important parameter to obtain the tumor response to chemotherapeutic agents, it represents the semi-inhibitory concentration of the agent and can be used to determine the tumor response to the agent, the lower the IC50 value indicates higher drug sensitivity and vice versa [29, 30]. Based on the Genomics of Cancer Drug Sensitivity (GDSC) database, drug sensitivity analysis was performed on each osteosarcoma sample using the R package pRRophetic, a model based on the relationship between baseline gene expression levels and in vitro cell line sensitivities to drugs, and ridge regression was used to predict the IC50 values of each sample for specific chemotherapeutic agents. The value of CRPS in guiding personalized treatment for osteosarcoma patients was also examined.

### Culture and transfection of cell lines

The osteosarcoma cell line SaoS-2 was purchased from American Type Culture Collection (ATCC). Cells were cultured in Dulbecco's Modified Eagle's Medium (DMEM) (5% CO_2_, 37°C) and the medium contained 10% fetal bovine serum (FBS), 100 IU/ml penicillin, and 100 mg/ml streptomycin. All reagents were purchased from Thermo Fisher.

### Gene transfection

RP11-414H17.5 was knocked down as si-RP11-414H17.5, abbreviated as siRNA (negative control as siRNA nc), overexpressed as PCMV-RP11-414H17.5-Neo, and abbreviated as RNAOE (negative control as RNAOE nc), and both were synthesized by Bacido Biotech Co. The constructed siRNA or plasmid was transfected into Saos-2 cells with Lipofectamine 2000 (Invitrogen, USA). si-RP11-414H17.5 target sequence was 5′-GAAGCTGCTTAAAGTGCAT-3′.

### Quantitative real-time PCR (qRT-PCR) detection

TRIzol reagent (Invitrogen, California, USA) was used to extract total RNA from tissues and cell lines, and equal amounts of RNA were then reverse-transcribed into cDNA using Prime Script RT kit (Takara, Dalian, China). SYBR Green kit (Takara) was used for qRT-PCR analysis. lncRNAs expression level was normalized using glyceraldehyde-3-phosphate dehydrogenase (GAPDH), and the relative expression of lncRNA was calculated using formula 2^(−∆∆CT)^. The primer sequences are shown in Table 1.

**Table 1:** The primer sequences of qRT-PCR

Gene	Primer sequence (5′-3′)
GAPDH(F)	GGAAGCTTGTCATCAATGGAAATC
GAPDH(R)	TGATGACCCTTTTGGCTCCC
RP11-414H17.5 (F)	GCACAGCTTCCCCGACTAAT
RP11-414H17.5 (R)	CACAGCGCGTGTTAGGAATG

### Detection of apoptosis by flow cytometry

The transfected cells were digested with trypsin and rinsed with pre-cooled PBS. The cells were resuspended in 1 × binding buffer with a concentration of 1 × 10^6 cells/mL. Apoptosis was detected using the Annexin V-FITC Apoptosis Detection Kit (Takara, Dalian, China). 5 μL of Propidium Iodide and 5 μL of Annexin V-FITC mixing were added to stain cells and they were incubated for 10 min at room temperature away from light before Saos-2 cell apoptosis was detected using flow cytometry.

### Wound-healing assay

The Saos-2 cells were inoculated at a density of 4 × 10^5 per well into a 6-well plate and incubated for 12 h. The wound was created by scratching the cell layer with a 200-μL sterile pipette tip. Serum-free nutrient solution was added after rinsing with PBS (Takara, Dalian, China). Imaging was performed at a 100 × field of view and the distance of wound closure was measured at 0, 24, and 48 h post-injury.

### Statistical analysis

This study performed statistical analysis using R software (version 4.2.3). Transcriptomic data and clinical data of all samples are available in the TARGET and GTEx databases (https://xena.ucsc.edu/). Correlations between different gene expressions were calculated using Spearman's correlation coefficient. Independent t test was used for analyzing the differences in quantitative data between different risk subgroups. The statistical differences criteria were set as P < 0.05.

## Results

### Screening of DECRLs

**Fig. 1:**
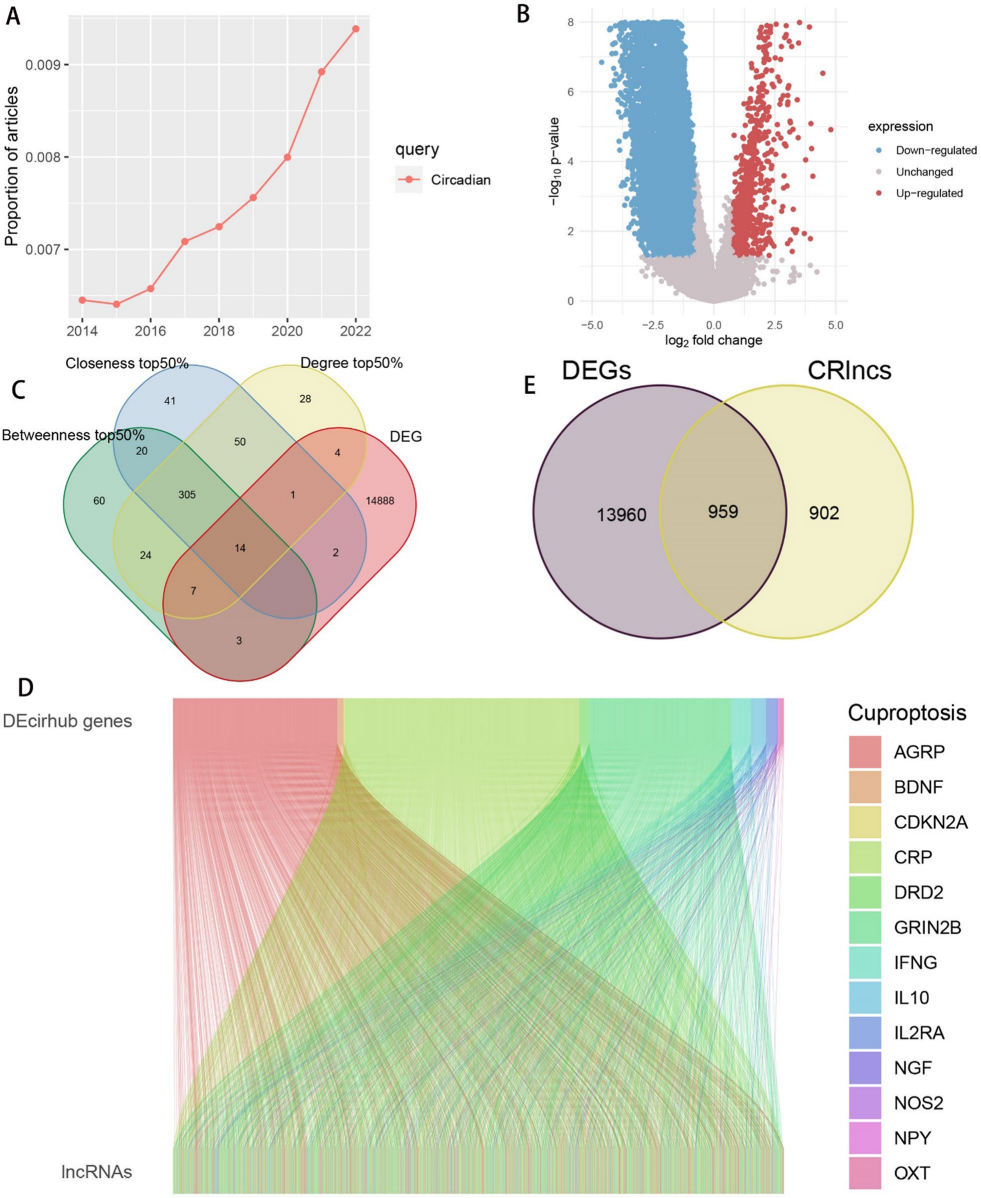
Acquisition of differentially expressed circadian rhythm-associated lncRNAs. **A** Recent research trends of circadian pathway; **B** DEGs between osteosarcoma and normal bone tissue samples; **C** Circadian-related genes of Betweenness, Closeness, and Degree—top 50% take intersection with DEGs; **D** Sankey diagram of the 14 hub genes with associated lncRNAs; **E** Venn diagram displaying the intersection between CRlncs and DEGs

The rising trend of research on the Circadian pathway in recent years can be seen in Fig. 1A. The DEGs between osteosarcoma and normal bone tissue samples are shown in Fig. 1B. The top 50% of circadian-related genes in Betweenness, Closeness, and Degree intersecting with DEGs were taken to get 14 hub genes (Fig. 1C). A total of 1861 CRlncs of the 14 hub genes were obtained by Pearson correlation analyses (Fig. 1D), with screening conditions of R > 0.4 and p < 0.001. A total of 959 DECRLs were then obtained by taking intersections with DEGs (Fig. 1E).

### Construction and verification of CRPS

**Fig. 2:**
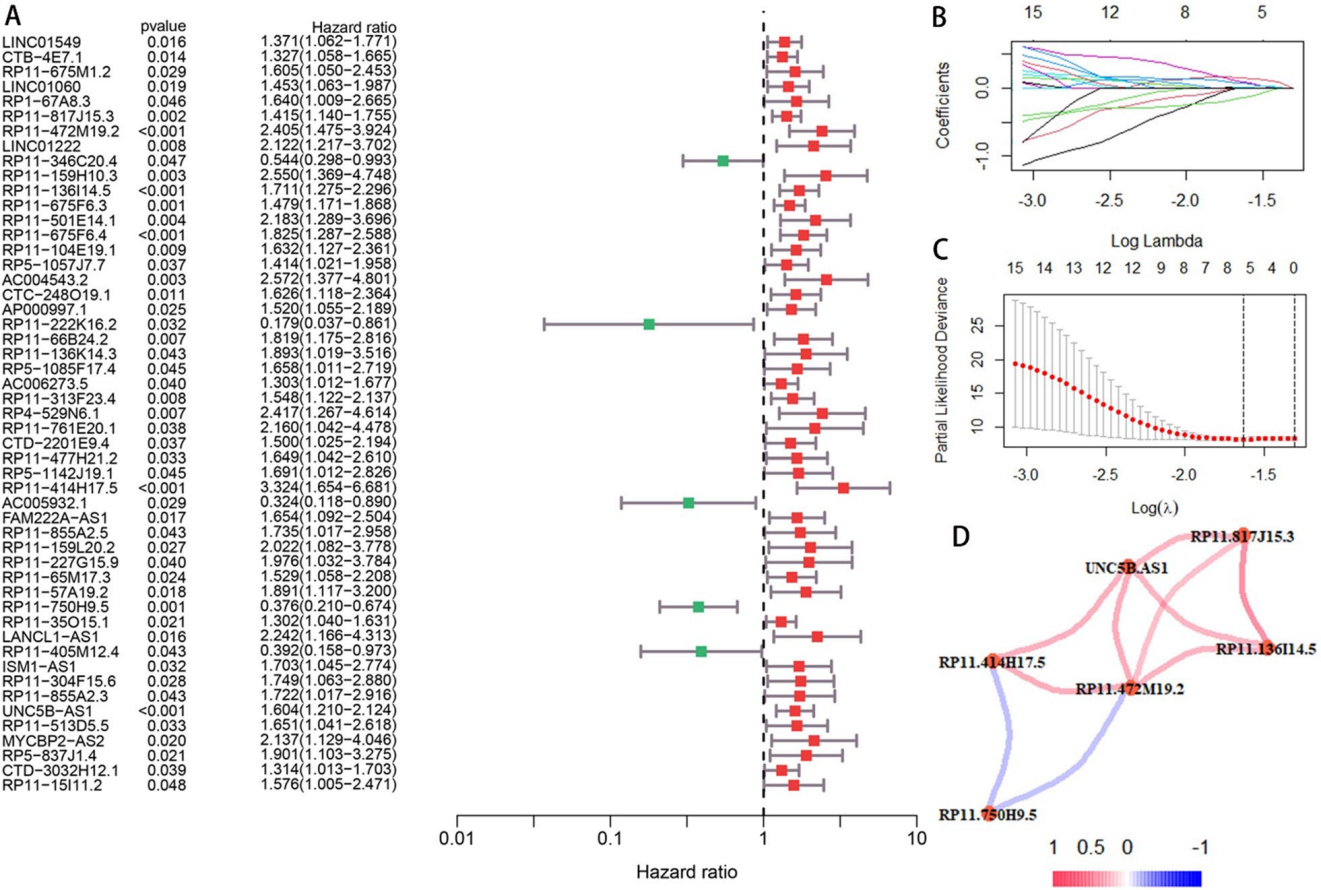
Constructing a prognostic prediction model CRPS. **A** Univariate COX regression analysis yielded 51 candidate DECRLs. **B**–**C** LASSO regression analysis yielded six prognostically relevant DECRLs. **D** Correlation node plots showing the correlation of the 6 DECRLs (red: positive correlations, blue: negative correlations)

**Fig. 3:**
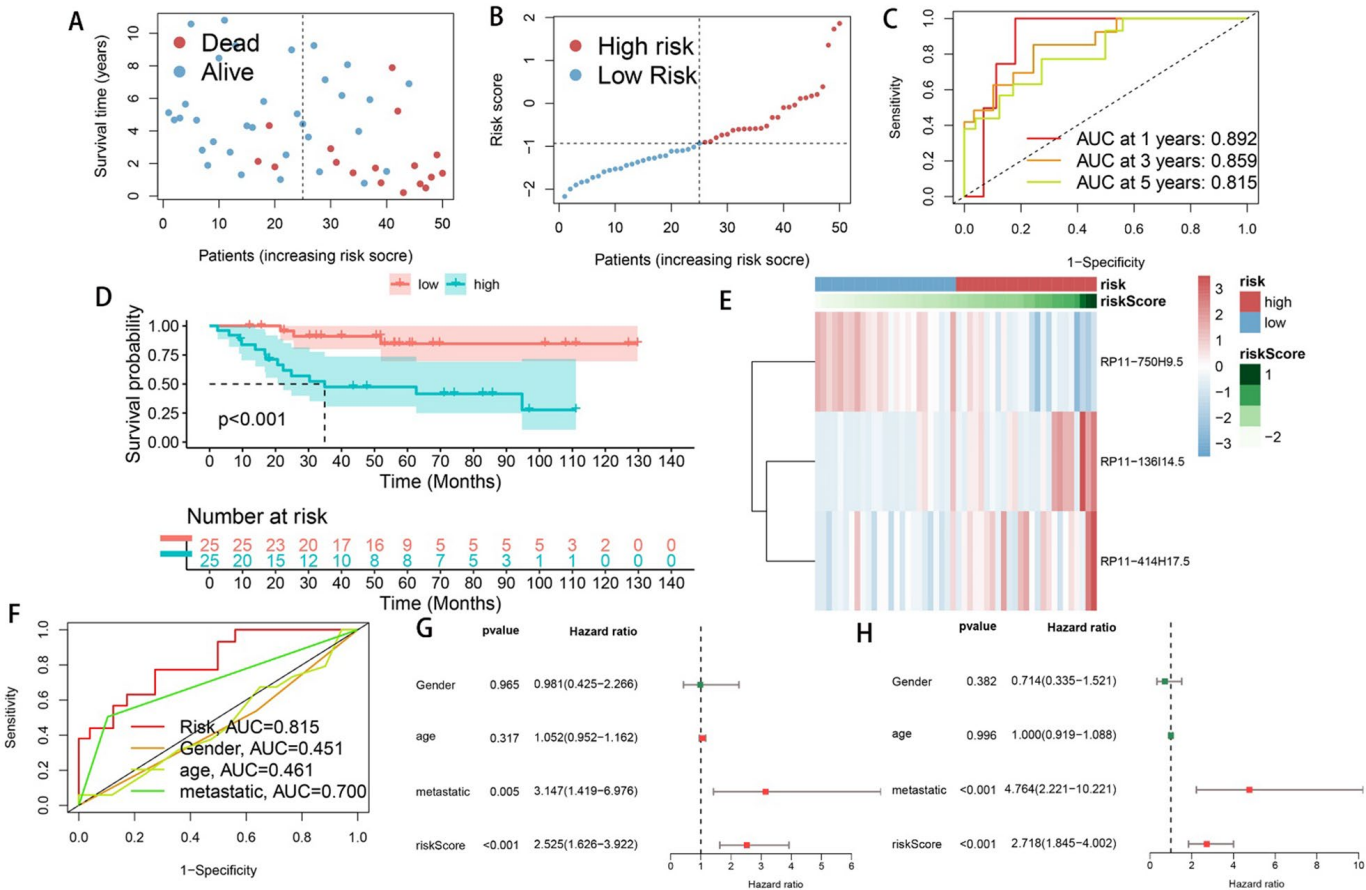
Prognostic predictive ability of CRPS assessed in the training group: **A**, **B** Scatter plots of survival in the training group; **C** 1-, 3-, and 5-year ROC plots of the training group; **D** KM curve in the training group; **E** Expression heat map of risk DECRLs in the training group; **F** Comparison of ROC curves of CRPS and different clinical characteristics in the training group; Univariate (**G**) and multivariate (**H**) forest plots of CRPS and different clinical features

Univariate COX regression was performed on 959 DECRLs and 51 prognostic-related candidate DECRLs were obtained (Fig. 2A). By performing LASSO regression analysis on the candidate DECRLs, six risk DECRLs were then derived (Fig. 2B, C). The correlation node network graph shows RP11.750H9.5 to be negatively correlated with the remaining five DECLRs (Fig. 2D). A multivariate COX regression analysis was performed on the six DECRLs, which yielded three DECRLs with the greatest prognostic value for the construction of a CRPS: RiskScore = (RP11-136I14.5 expression)*0.3174167 + (RP11-414H17.5 expression)* 0.5083453-(RP11-750H9.5 expression)*0.6138377. Each osteosarcoma sample received a risk score that was determined by CRP and they were then all classified into different risk subgroups based on median value. Follow-up analysis was then performed as a means of verifying the ability of CPRS to independently predict prognosis. Scatter plots and heatmaps of survival status, ROC curves, ROC curves, and KM curves show CRPS to be an effective predictor for forecasting osteosarcoma patient prognosis (Fig. 3A–F). The AUC values of CRPS for predicting the 1-, 3-, and 5-year prognosis reached 0.892, 0.859, and 0.815, respectively, and the AUC value of CRPS far exceeded those of other clinical features, which indicates that CRPS had the strongest predictive ability. The different CRPS-classified risk subgroups showed significant differences in terms of survival. Univariate and multivariate forest plots (Fig. 3G, H) show that CRPS and metastasis independently predicted osteosarcoma prognosis.

### Verify the stability of CRPS

**Fig. 4:**
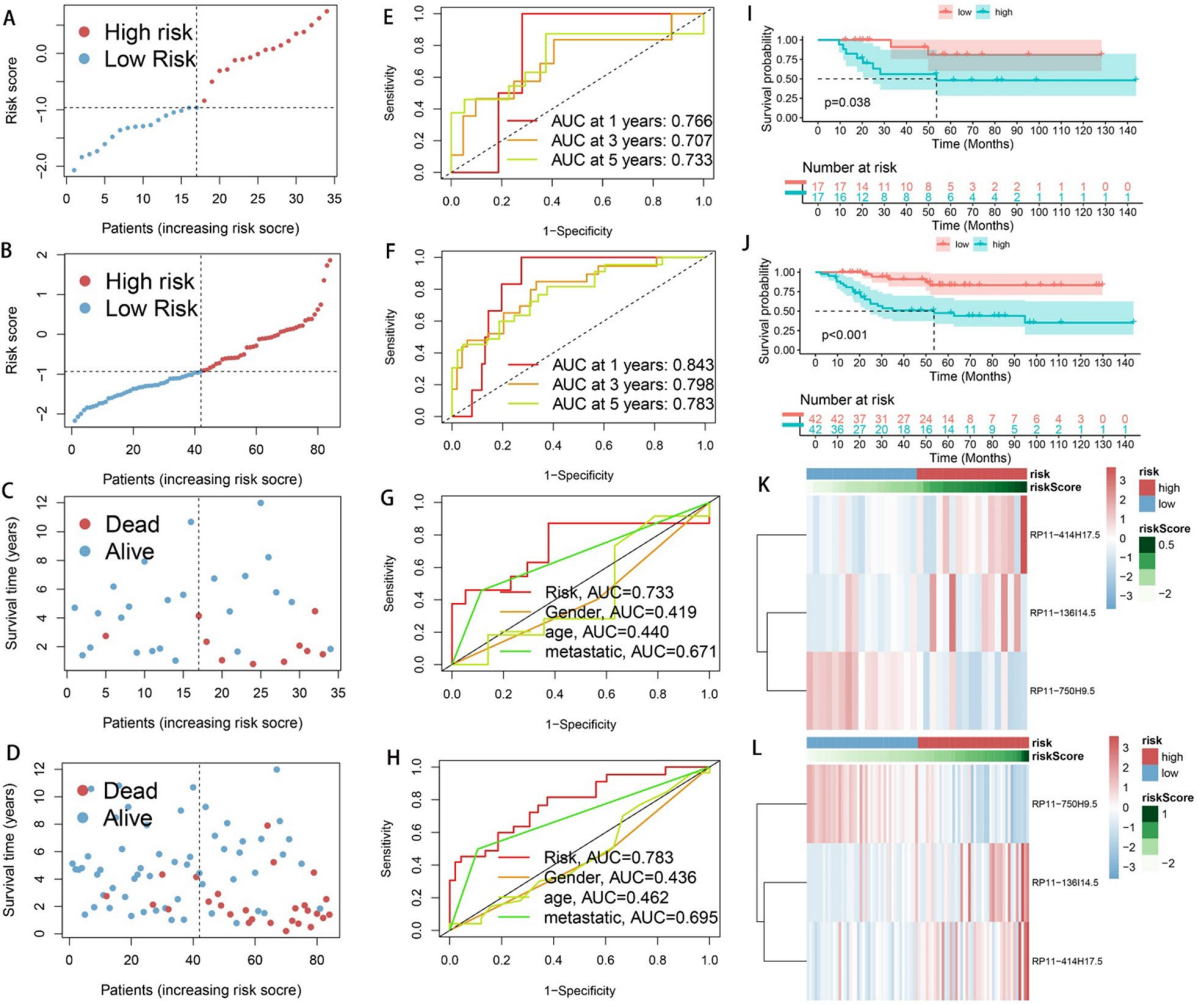
Validation of the prognostic predictive power of CRPS. Scatter plots of patient survival status in test group (**A**, **C**) and all cohorts (**B**, **D**). 1-, 3-, and 5-year ROC curves in test set (**E**) and all cohort (**F**). ROC curves of CRPS and clinical features were plotted and compared in test set (**G**) and all cohorts (**H**), AUC values of CRPS were consistently superior to other clinical features. Significant differences in survival status across risk subgroups were showed by KM curves in test group (**I**) and all cohort (**J**). Heatmaps of the risk DECRLs expression of test group (**K**) and all cohort (**L**)

**Fig. 5:**
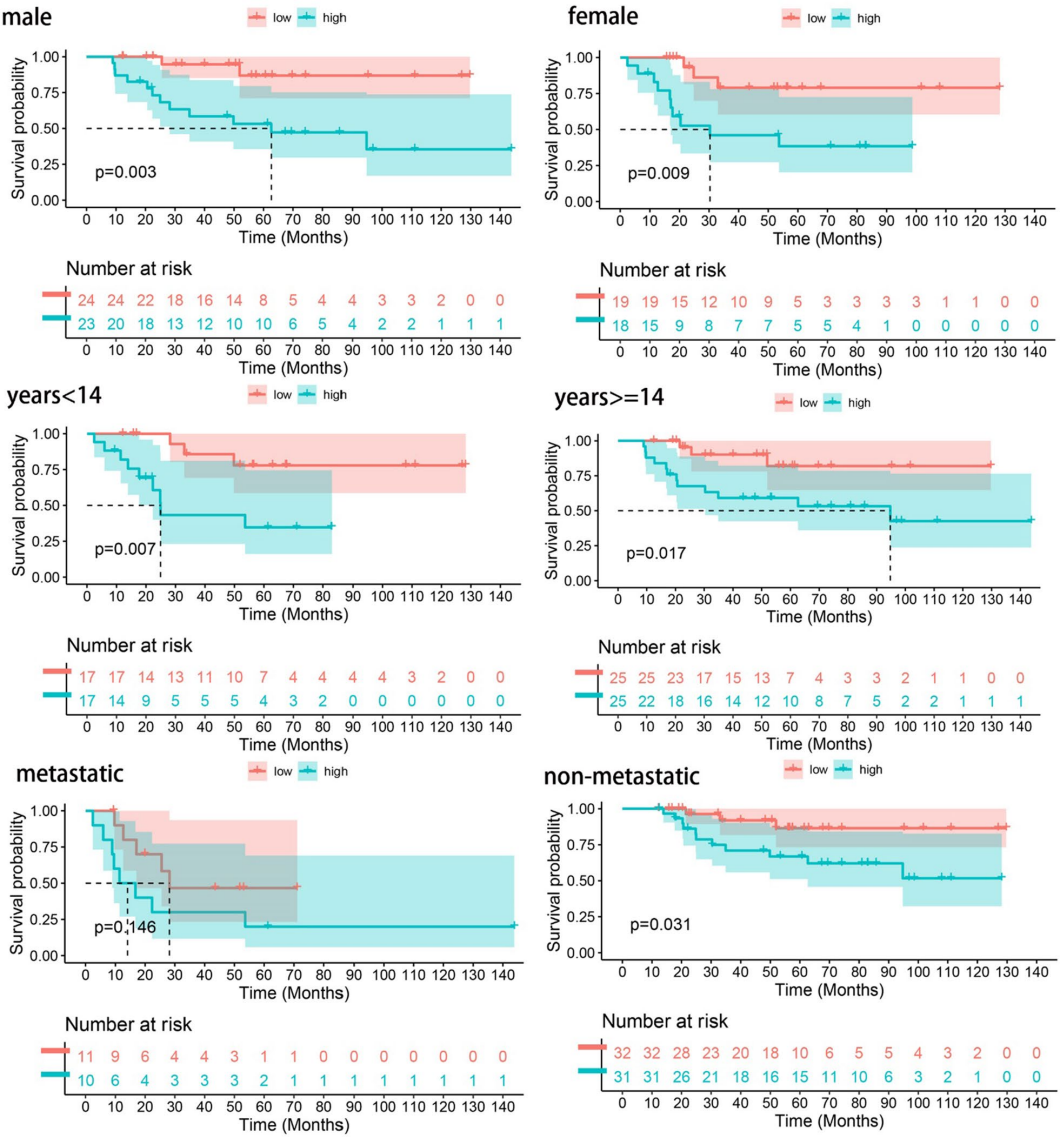
Regrouping patients according to their clinical traits (age, gender, whether metastatic or not) and plotting KM curves, then observing the performance of the risk model in different clinical subgroups

**Fig. 6:**
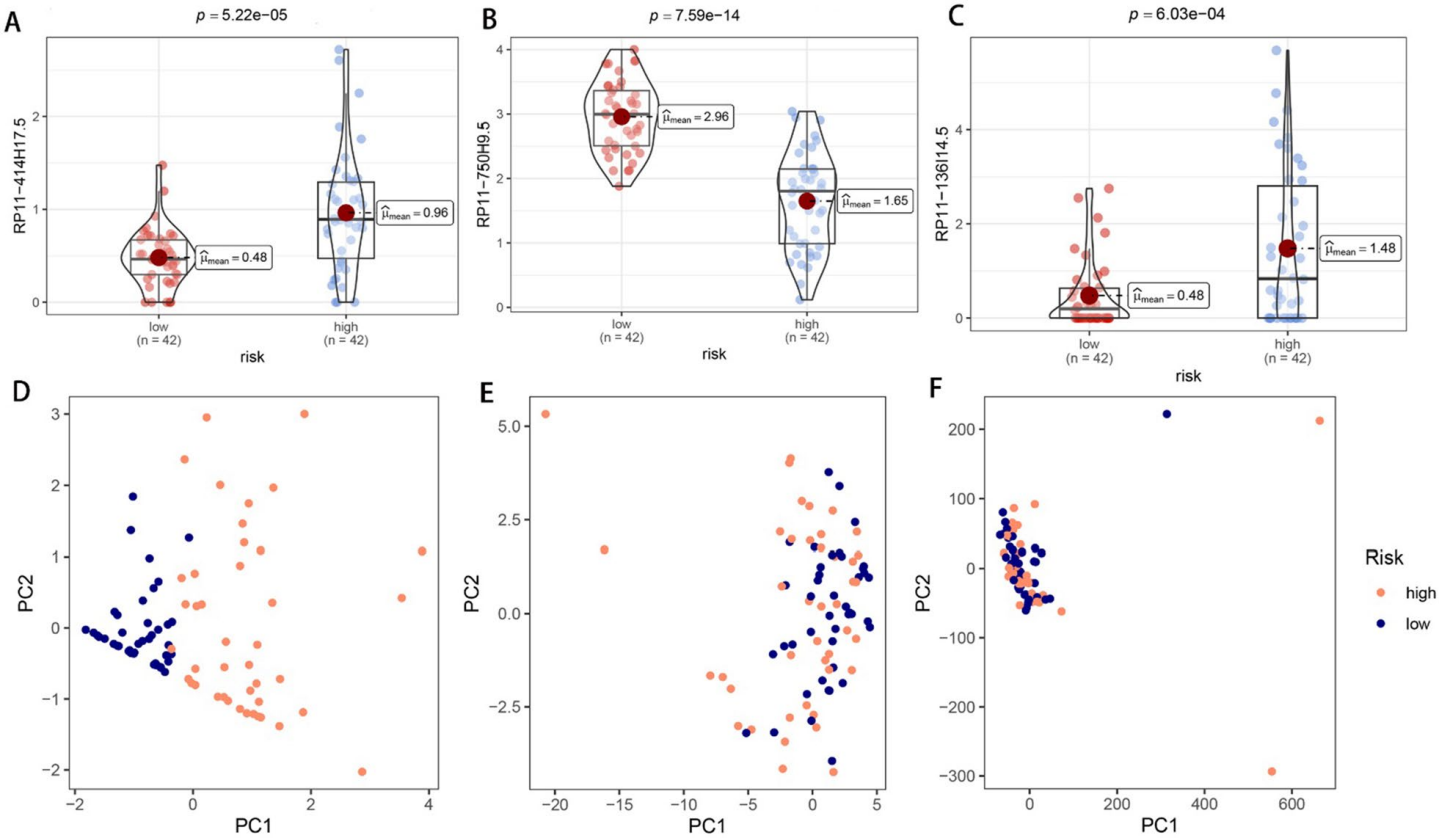
Performing PCA and DECRLs box plot analysis in all cohorts. The box plots showed that the expression of RP11-414H17.5 (**A**), RP11-750H9.5 (**B**), and RP11-136I14.5 (**C**) were significantly different between different risk subgroups. PCA results indicated that these 3-risk DECRLs could significantly distinguish between the different risk subgroups (**D**), whereas the 51 candidate DECRLs (**E**) and all the genes (**F**) were less effective

The same analyses as in Fig. 3 were conducted for the test set and all cohorts in order to examine CRPS stability. The high-risk group showed a significantly higher mortality rate, as can be seen in Fig. 4A–D. The AUC values reached 0.766, 0.707, and 0.733 at 1-, 3-, and 5-year in the test group, while they were 0.843, 0.798, and 0.783 in all cohorts, as shown in Fig. 4E–F. Figure 4G–H shows that the AUC values of CRPS were superior to other clinical features in the test group and all cohorts. The KM curves show significant differences in survival across risk subgroups in the test group and all cohorts. The risk heat map indicates that RP11-136I14.5 and RP11-414H17.5 were highly expressed in the high-risk group, while RP11-750H9.5 had low expression. As shown in Fig. 5, there were significant differences in survival between risk subgroups, except for the metastatic patient group. Figure 6A–C shows that the expression of 3-risk DECLRs between different risk subgroups was significantly different. The PCA result indicated that the 3-risk DECLR provides a clear separation of different risk subgroups into two clusters (Fig. 6D), whereas none of the 51 candidate DECLRs, and all the genes, could clearly classify the different risk subgroups (Fig. 6E, F).

### Constructing a nomogram

**Fig. 7:**
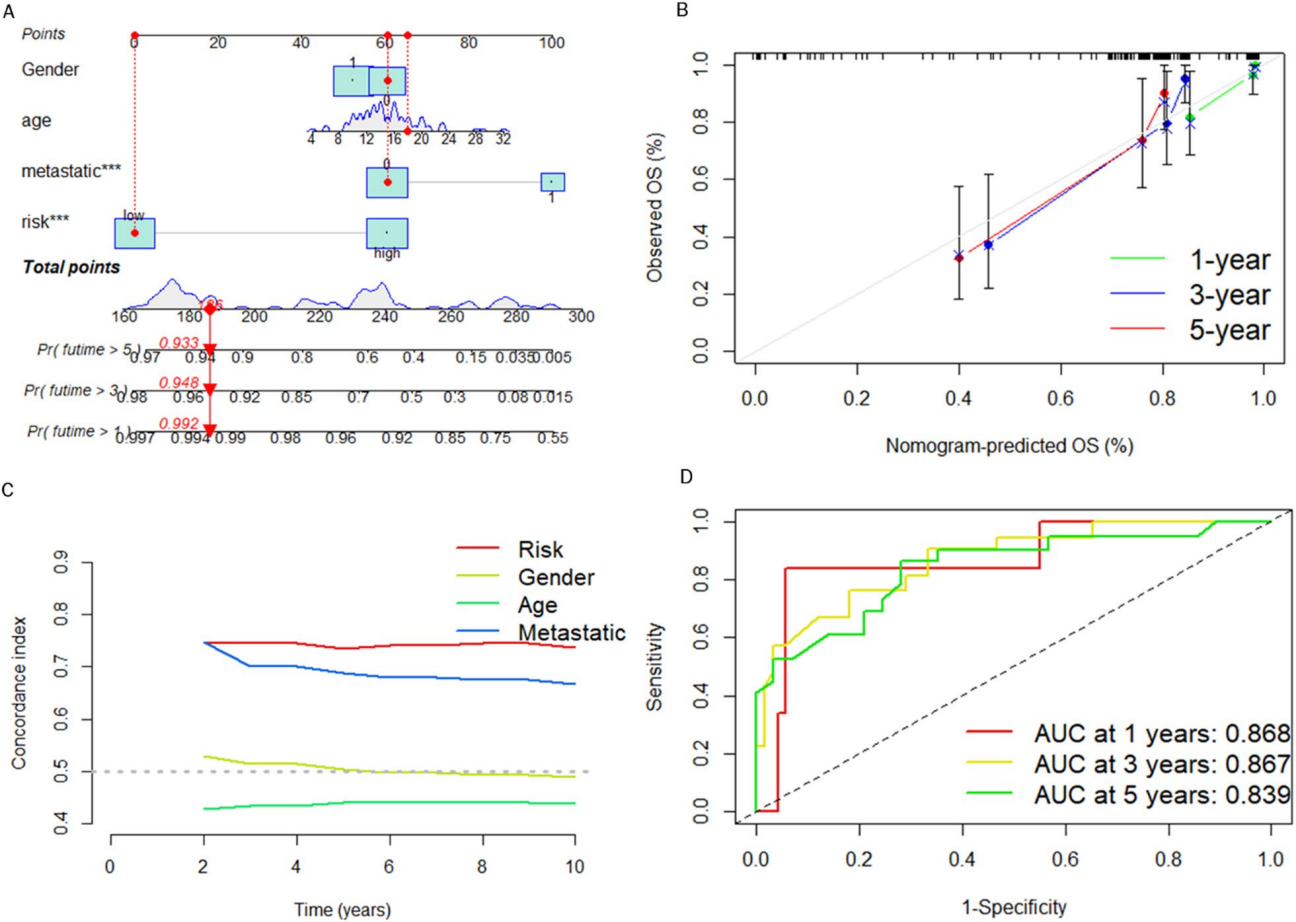
Constructing a nomogram for predicting survival of osteosarcoma patients. **A** Constructed nomogram based on CRPS and clinical features. **B** Calibration curve showing the consistency between predicted values and actual values. **C** C-index curve shows that LRPS predicts prognosis better than other clinical characteristics. **D** ROC curves predicting prognosis of osteosarcoma patients based on nomogram predictive values

In an attempt to improve the prediction of survival probability of osteosarcoma patients at different time points, a nomogram was constructed that combined CRPS and other clinical features (Fig. 7A). The calibration curves show that the predictions of this nomogram matched the actual situations to a high degree (Fig. 7B). The C-index curve shows that CRPS best distinguished osteosarcoma patient prognosis (Fig. 7C). An ROC curve was plotted and the AUC value was calculated based on the predicted values that were obtained from the nomogram. The results show that the AUC was up to 0.868 (Fig. 7D), which confirms that this nomogram could be a good predictor of survival for osteosarcoma patients.

### Biological pathway enrichment analysis

**Fig. 8:**
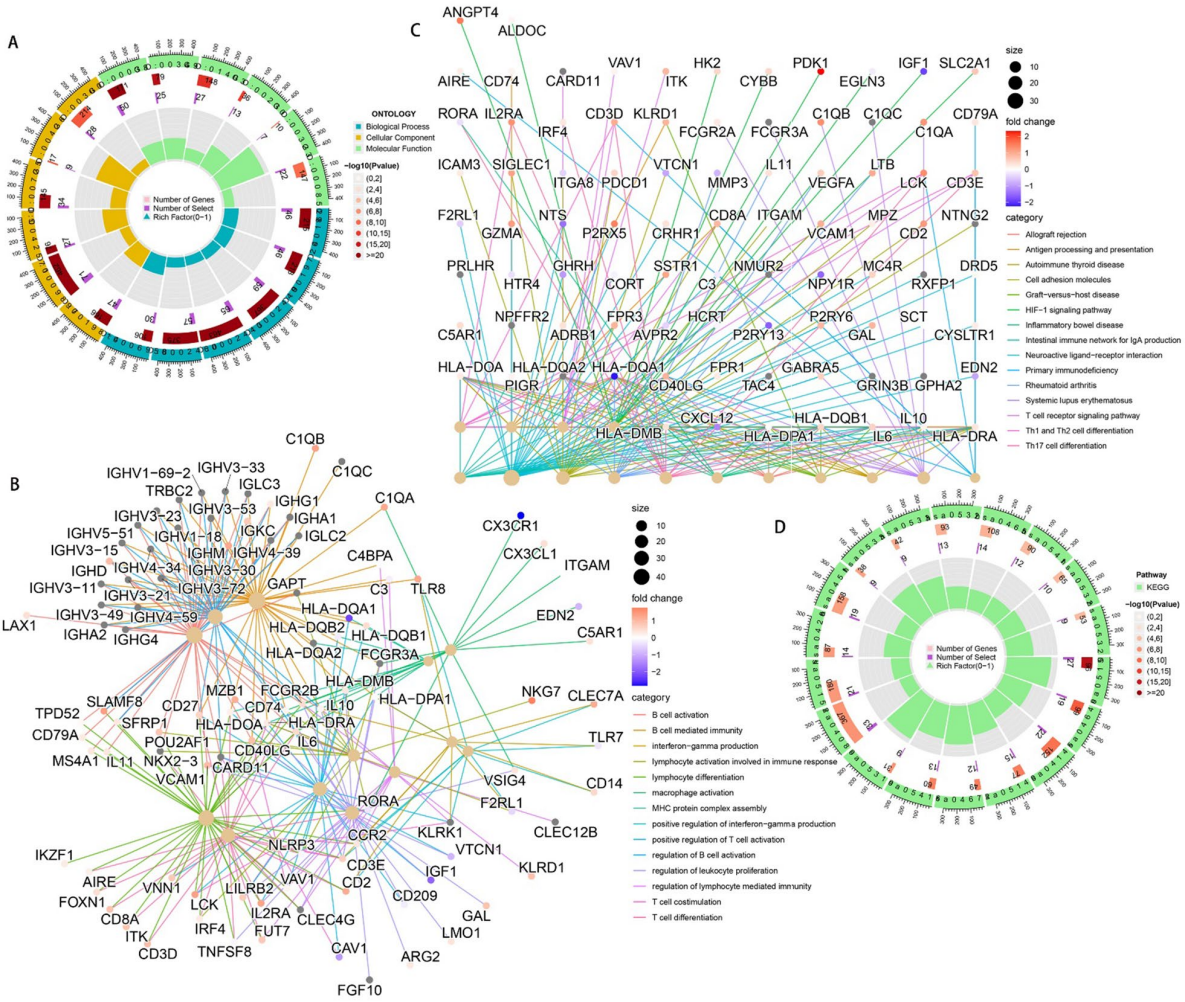
Enrichment analysis results of DEGs between different risk subgroups to observe potential pathways of promoting disease progression by CRPS. **A**, **B** GO pathway enrichment analyses result. **C**, **D** KEGG pathway enrichment analyses result

To explore the relevant pathways by which CRPS affects the prognosis of osteosarcoma, differential expression analysis was performed on different risk subgroups and 1,445 DEGs were obtained. GO enrichment analyses were conducted on these DEGs and the results can be seen in Fig. 8A, B. These genes were significantly enriched in pathways such as T cell differentiation, B cell activation, lymphocyte activation involved in immune response, and macrophage activation. Similarly, KEGG enrichment analysis was performed on these genes, the results of which are shown in Fig. 8C, D. These genes were significantly enriched in pathways such as Th17 cell differentiation, cell adhesion molecules, Th1 and Th2 cell differentiation, antigen processing and presentation, T cell receptor signaling pathway, and primary immunodeficiency. It is suggested that CRPS may have an impact on osteosarcoma progression through the above pathways.

### Probing the effects of CRPS on immune infiltration and immune checkpoints

**Fig. 9:**
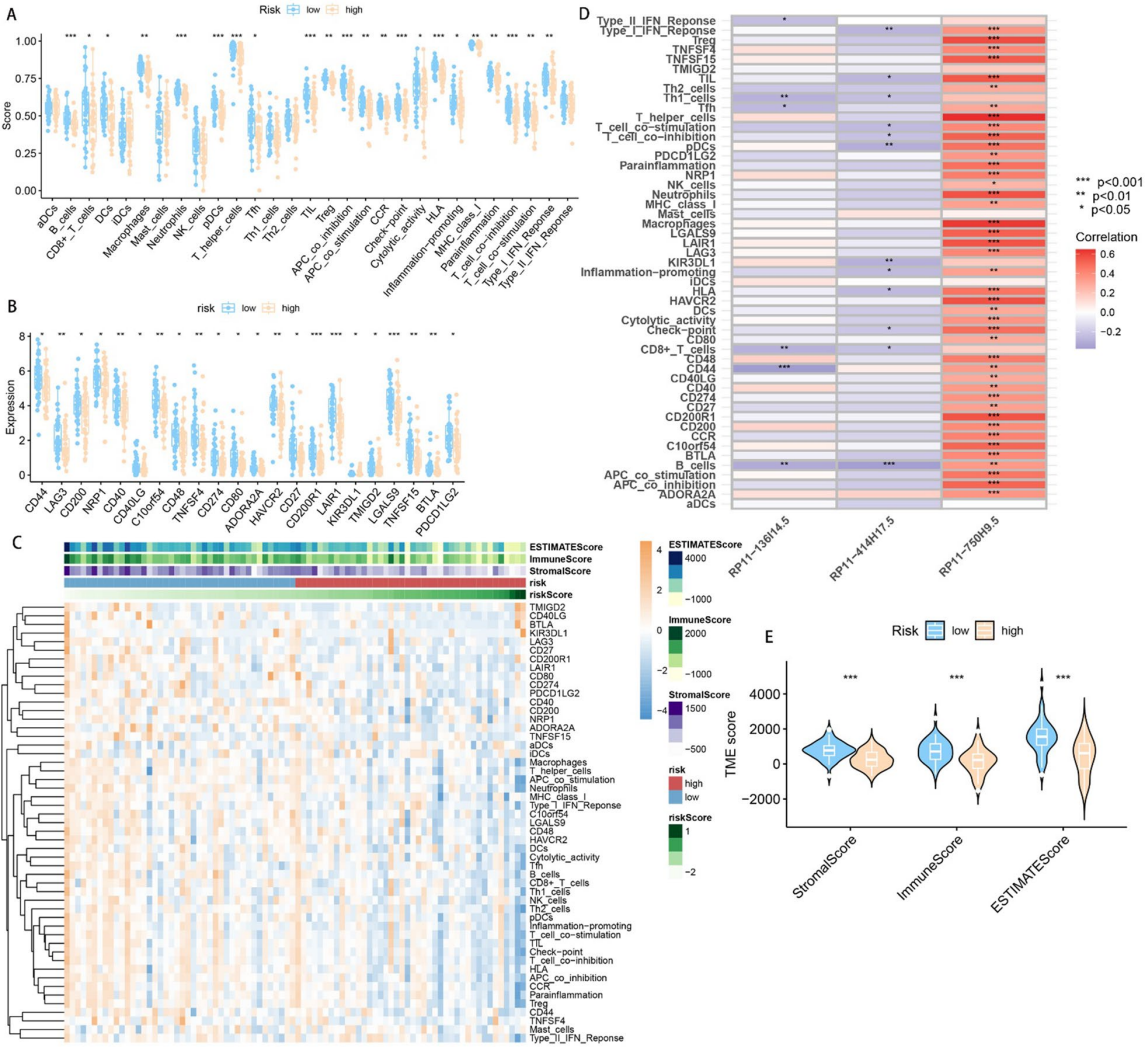
Immune checkpoints and Immune infiltration analyses based on CRPS. **A** Box plots of immune cells and immune function scores of all osteosarcoma samples, compared with high-risk group, the low-risk group has better Immunescores. **B** Immune checkpoint expression was higher in the low-risk group compared with the high-risk group. **C** Heatmap of osteosarcoma immune infiltration in different risk subgroups, immune infiltration was better in the low-risk group. **D** A correlation heatmap showed a positive correlation between RP11-750H9.5 and immune infiltration, while RP11-136I14.5 and RP11-414H17.5 were negatively correlated with immune infiltration. **E** Violin plot shows that ESTIMATEscore Immunescore and Stromalscore were significantly lower in the high-risk group. (*, **, *** indicate P < 0.05, P < 0.01, P < 0.001, respectively.)

**Fig. 10:**
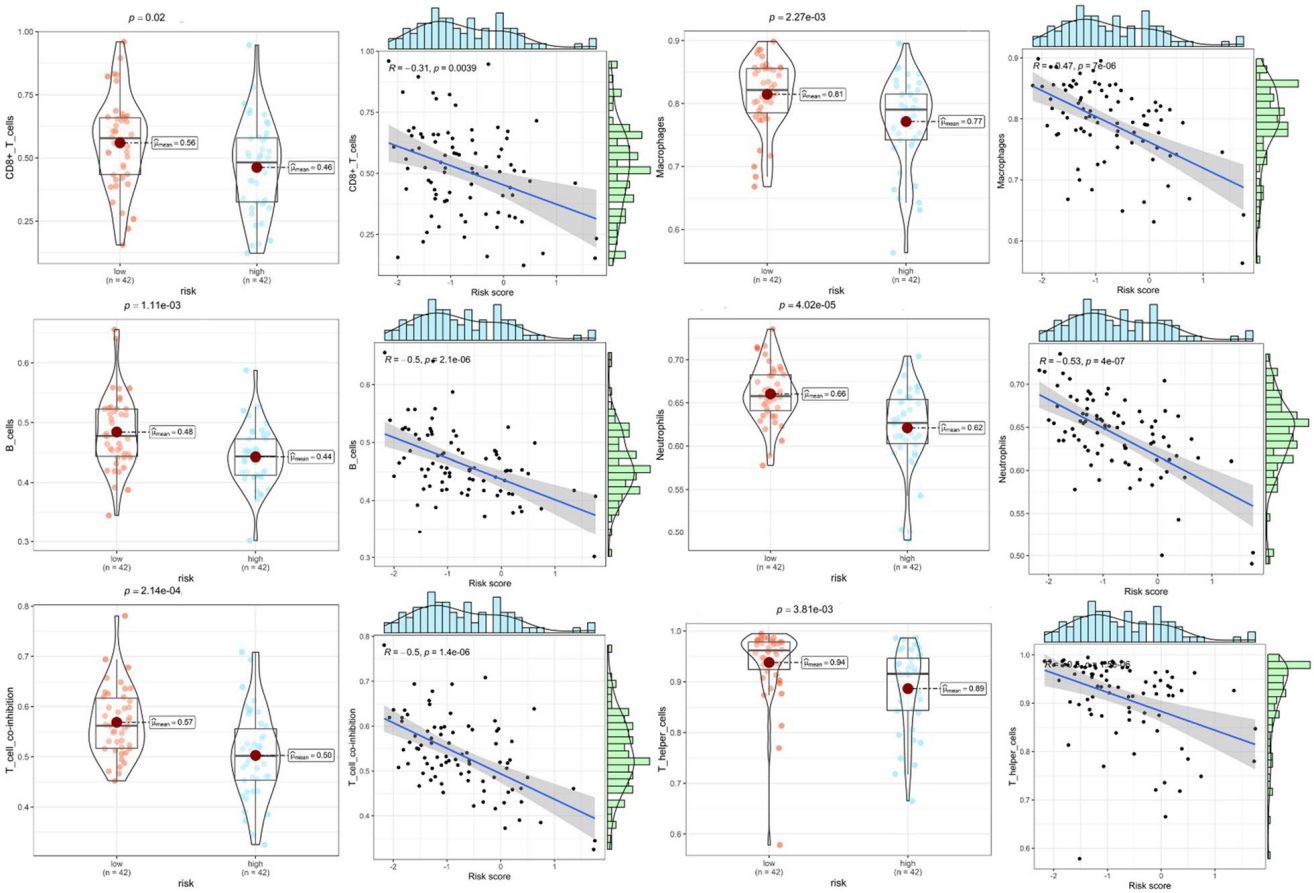
Analysis of the correlation between CRPS and immunological infiltration. Differences in immunological infiltration between two risk subgroups are shown in box plots, while the association between immunological infiltration and risk scores is shown in correlation scatter plots

**Fig. 11:**
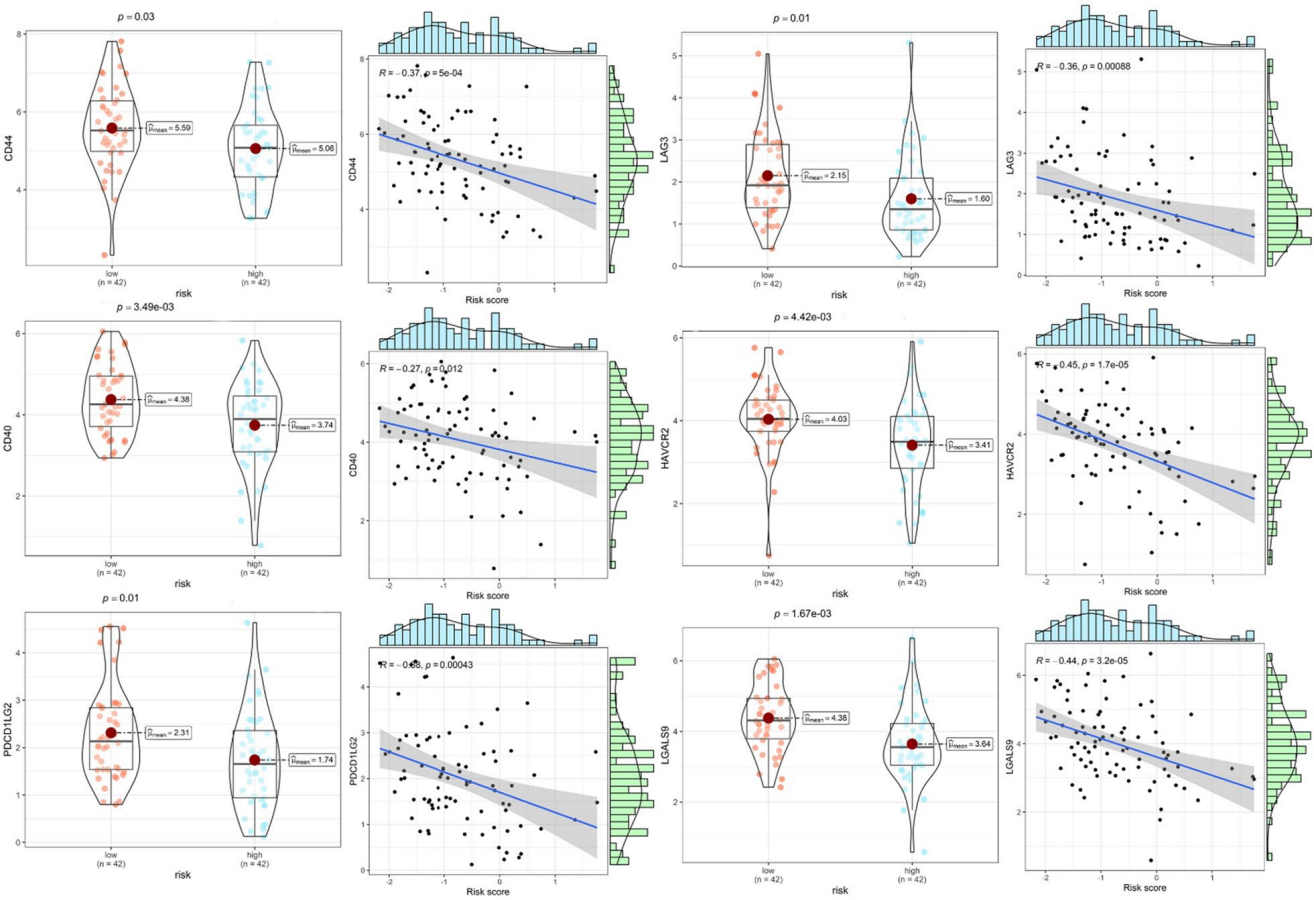
Analysis of the association between immune checkpoints and CRPS. Box plots show differences in immune checkpoints expression between different risk subgroups, as well as correlation scatter plots show the correlation between risk scores and immune checkpoints expression

To probe the potential association between CRPS and the immune microenvironment of osteosarcoma, immune infiltration analyses were conducted on osteosarcoma samples using the R package GSVA. Figure 9A shows that there are significant disparities in immune infiltration conditions between risk subgroups. Immune infiltration was found to be better in the low-risk group, as reflected in immunological scores such as macrophages, CD8 + T cells, B cells, and T cell co-stimulation. Figure 9B shows that immune checkpoints including CD44, CD40, and PDCD1LG2 had high expression in the low-risk group. The immunological heat map demonstrates that the immune infiltration status was significantly better in the low-risk group (Fig. 9C). The correlation heatmap shows that RP11-750H9.5 was positively and significantly associated with immune infiltration, while the opposite was observed for RP11-136I14.5 and RP11-414H17.5 (Fig. 9D). Violin plots show there to be significant differences in Immunescore, Stromalscore, and ESTIMATEscore in different risk subgroups. Correlation scatter plots and box line plots show the correlation of CRPS with different immune cells, immune functions, and immune checkpoints (Fig. 9). These results all serve to indicate that CRPS can significantly affect the immune microenvironment of osteosarcoma, in addition to being applicable for the guidance of osteosarcoma immunotherapy (Figs. 10, 11).

### Chemotherapeutic drug susceptibility analyses

**Fig. 12:**
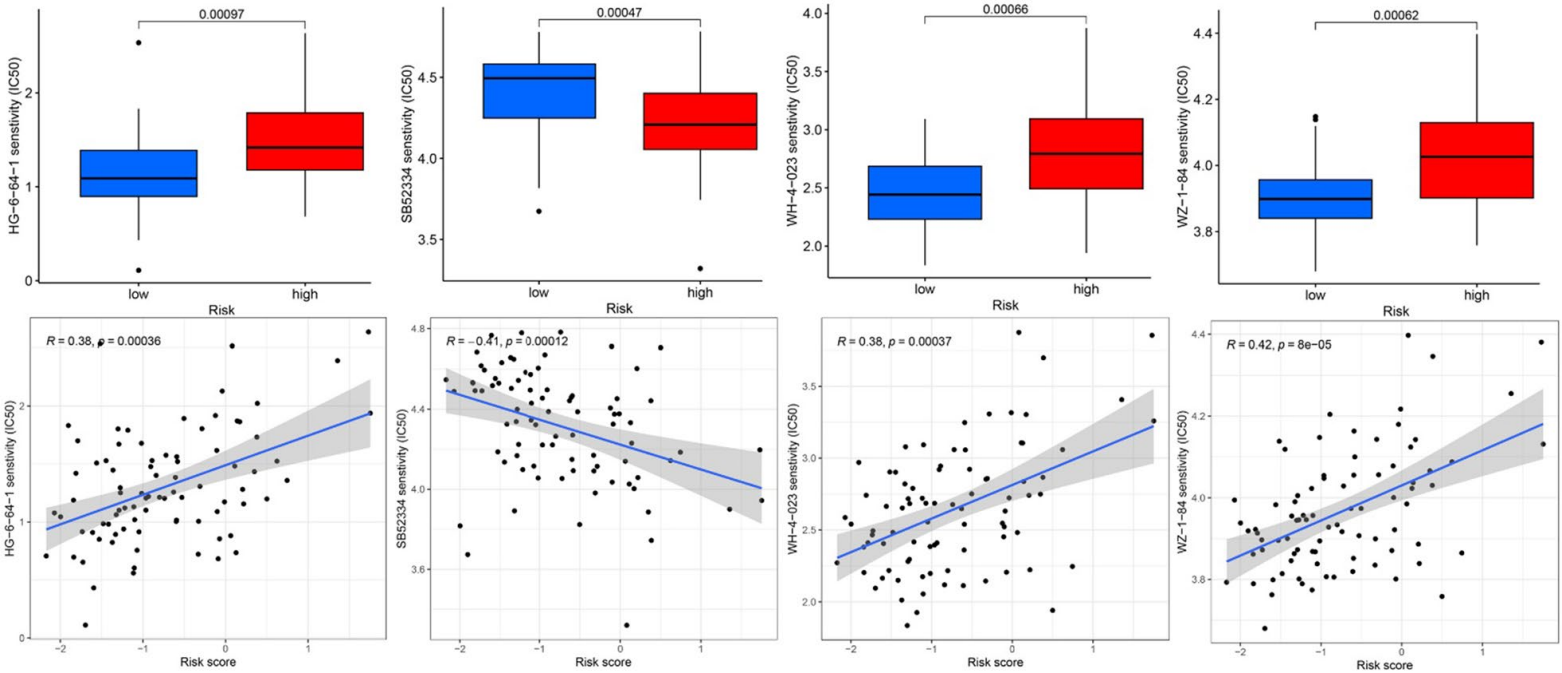
Chemotherapy drug sensitivity analysis to guide individualized treatment of osteosarcoma patients. Box plots showed lower IC50 values for drugs HG-6-64-1, WH-4-023, and WZ-1-84 in the low-risk group, as well as the correlation scatter plots displayed that risk scores were positively correlated to IC50, suggesting that the low-risk group patients would be more susceptible toward these drugs. The opposite was observed for SB52334, suggesting that the high-risk group patients would be more susceptible toward this drug

In an attempt to further probe the disparity of sensitivity to agents between different risky subgroups, box plots were used to compare the IC50 values of both groups, in addition to plotted correlation scatter diagrams between IC50 value and risk score. The results are presented in Fig. 12. The lower IC50 values of HG-6-64-1, WH-4-023, and WZ-1-84 in the low-risk group suggest this group would be more susceptible to these agents. The opposite was observed for SB52334, which indicates the better sensitivity of the high-risk group to this drug. The results all suggest that CRPS can provide guidance for individualized drug therapy in osteosarcoma patients.

### Probing the relationship between DECRLs and osteosarcoma metastasis

**Fig. 13:**
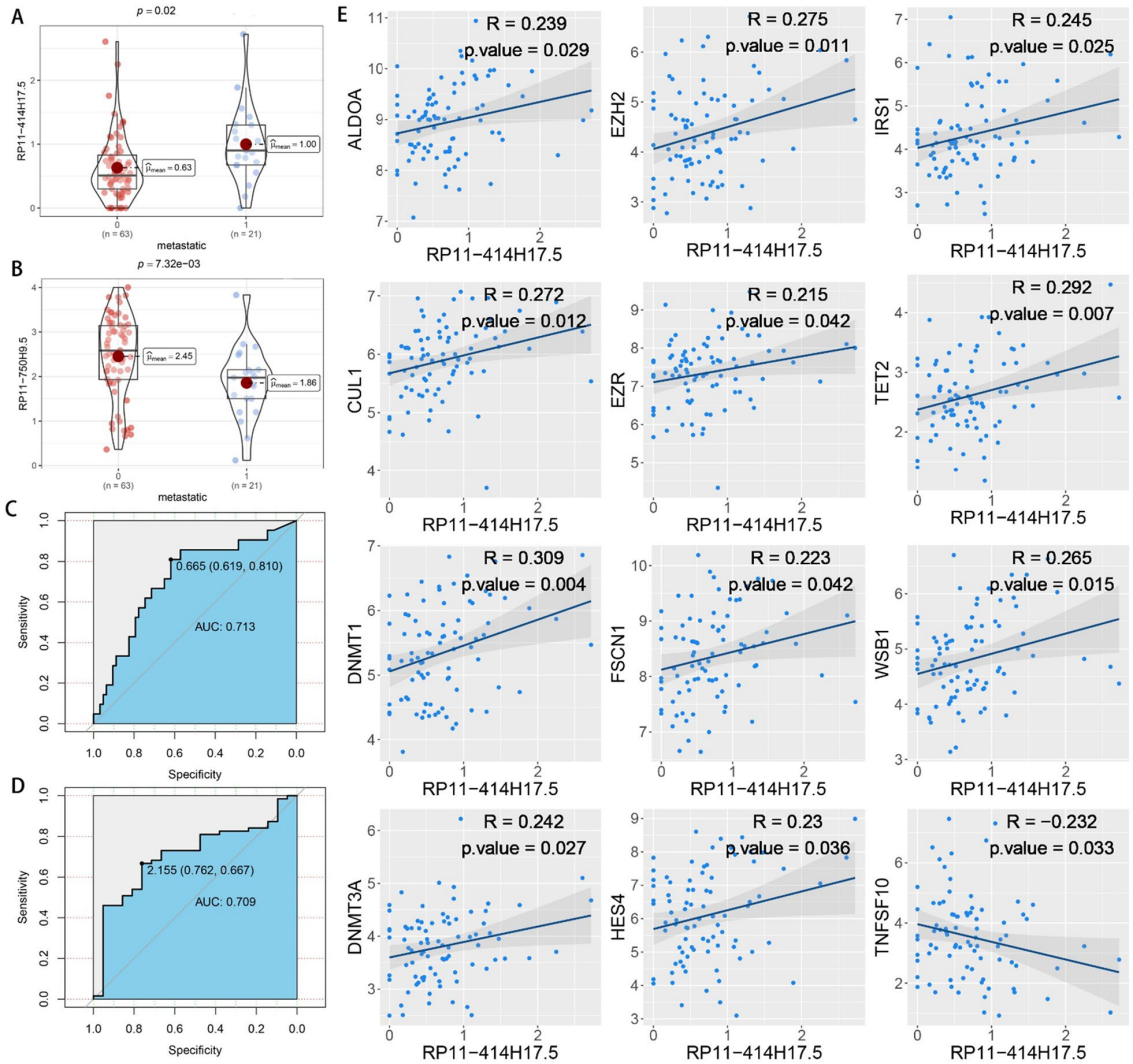
Correlation analysis of DECRLs with osteosarcoma metastasis. Box plots showed that RP11-414H17.5 (**A**) and RP11-750H9.5 (**B**) were differentially expressed in different metastatic subgroups. The ROC curves showed that RP11-414H17.5 predicted osteosarcoma metastasis with an AUC value of 0.713 (**C**), and RP11-750H9.5 predicted non-metastasis of osteosarcoma with an AUC value of 0.709 (**D**). Correlation scatter plots display the association between RP11-414H17.5 and osteosarcoma metastasis-related genes (**E**)

Box diagrams and ROC curves were plotted to probe the potential association between DECRLs and osteosarcoma metastasis. The results showed both RP11-414H17.5 and RP11-750H9.5 to be differentially expressed in different metastatic subgroups (Fig. 13A, B). RP11-414H17.5 predicted osteosarcoma metastasis with an AUC value up to 0.713 (Fig. 13C), while RP11-750H9.5 predicted the non-metastasis of osteosarcoma with an AUC value up to 0.709 (Fig. 13D). The results suggest that RP11-414H17.5 potentially promotes osteosarcoma metastasis. The correlation between RP11-414H17.5 and osteosarcoma metastasis-related genes was then analyzed and the results can be seen in Fig. 11E. It was demonstrated that RP11-414H17.5 had a positive correlation with osteosarcoma metastasis-promoting genes, e.g., ALDOA, EZH2, IRS1, etc., and negatively correlated with the gene TNFSF10, which inhibits osteosarcoma metastasis. These results strongly suggest the promoting effect of RP11-414H17.5 on osteosarcoma metastasis.

### Biological pathway enrichment analysis of RP11-414H17.5

**Fig. 14:**
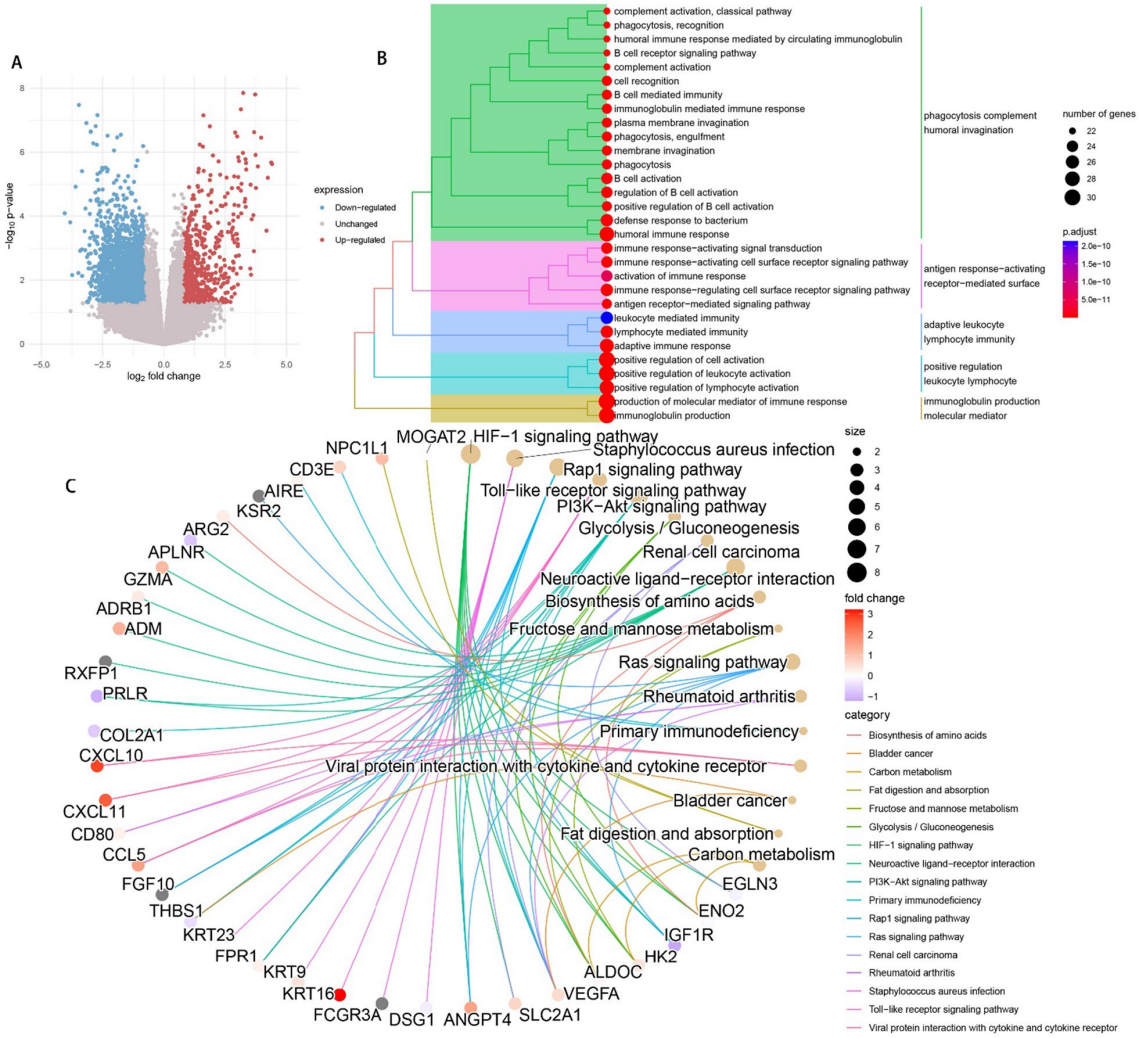
Biological pathway enrichment analysis of differentially expressed genes between different risk subgroups, to explore potential pathways associated with CRPS. (**A**) Volcano map of DEGs between different risk subgroups. (**B**) The GO enrichment analyses results. (**C**) The KEGG enrichment analyses results

To explore the potential pathways involved in RP11-414H17.5 affecting osteosarcoma prognosis, low- and high-expression subgroups were classified based on RP11-414H17.5 expression and differential expression analysis was performed. Figure 14A shows the volcano plot of DEGs. GO enrichment analysis was subsequently conducted on 310 DEGs, as shown in Fig. 14B. DEGs were found to be significantly enriched in B cell receptor signaling pathway, complement activation, leukocyte-mediated immunity, immunoglobulin production, B cell activation, and many other immune-related pathways. KEGG enrichment analysis of DEGs showed significant enrichment of these genes in HIF-1 signaling pathway, PI3K-Akt signaling pathway, Ras signaling pathway, bladder cancer, renal cell carcinoma, and other pathways. It is suggested that RP11-414H17.5 could affect tumor progression through the above molecular pathways, in addition to being associated with bladder cancer and renal cell carcinoma development (Fig. 14C).

### RP11-414H17.5 promotes migration and inhibits apoptosis of osteosarcoma cells

**Fig. 15:**
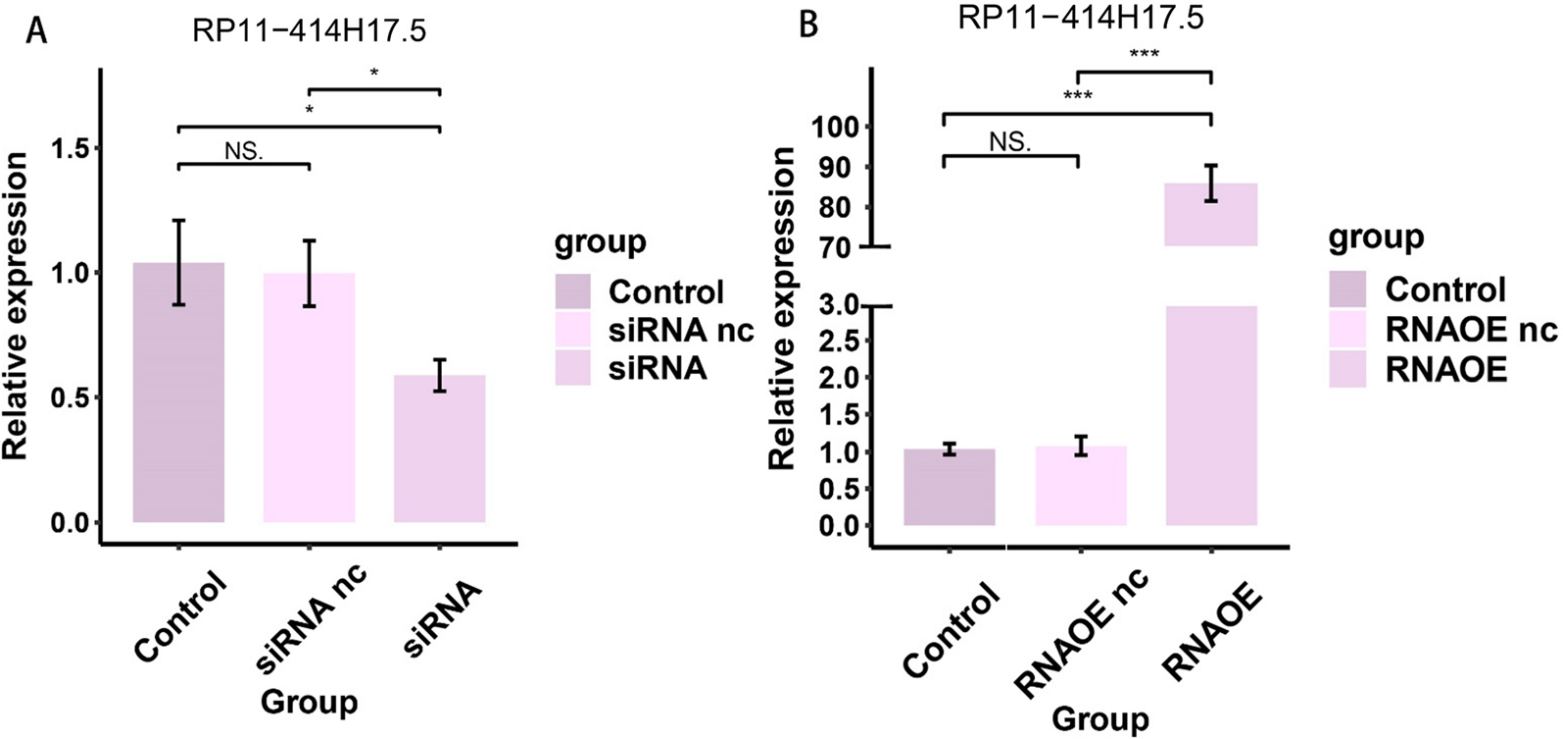
qRT-PCR experiment results of Saos-2 cell lines. (**A**) Successful knockdown of RP11-414H17.5 in Saos-2 cells. (**B**) Successful overexpression of RP11-414H17.5 in Saos-2 cells

**Fig. 16:**
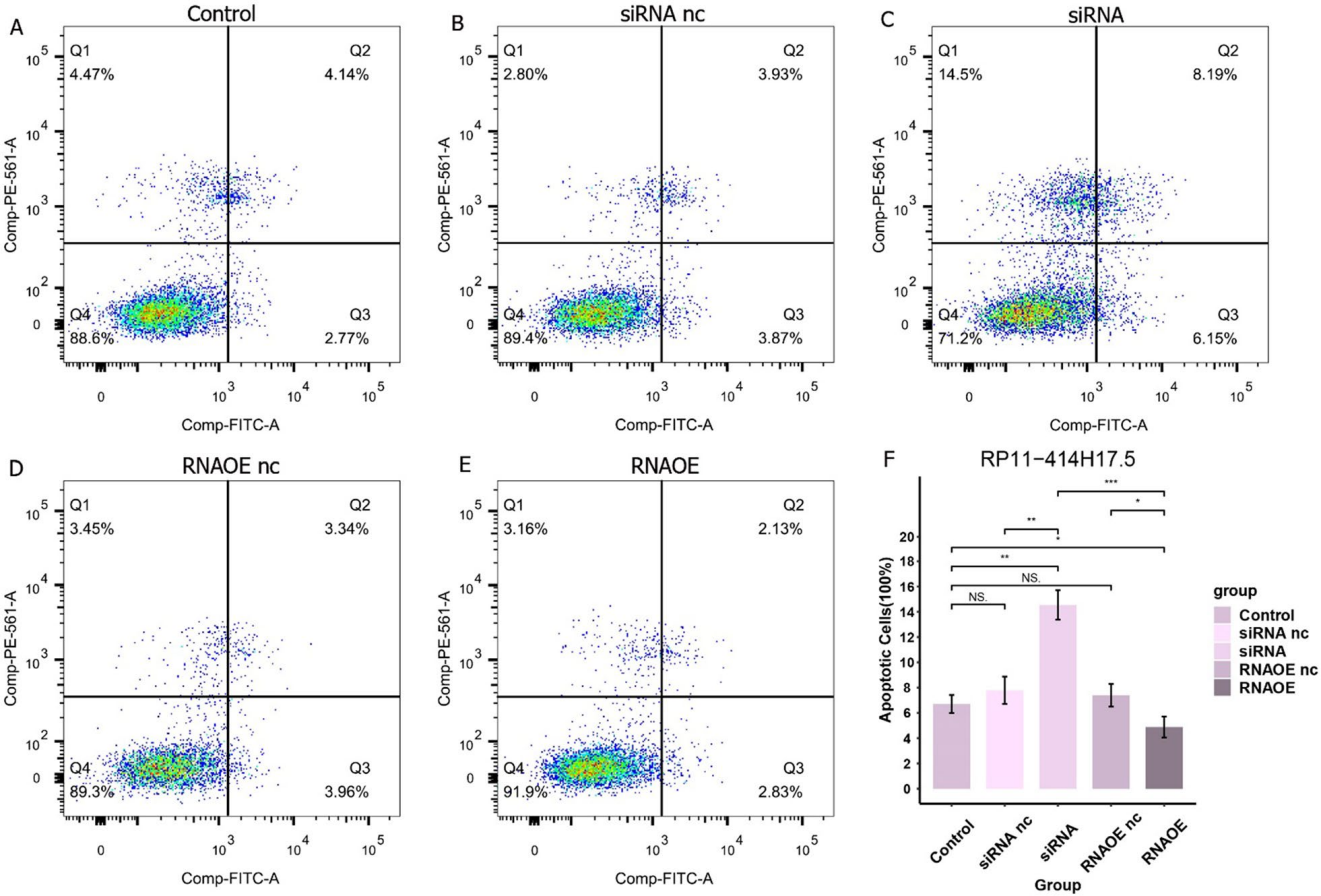
Knockdown of RP11-414H17.5 significantly promoted apoptosis in Saos-2 cells, and overexpression of RP11-414H17.5 significantly inhibited apoptosis in Saos-2 cells.

**Fig. 17:**
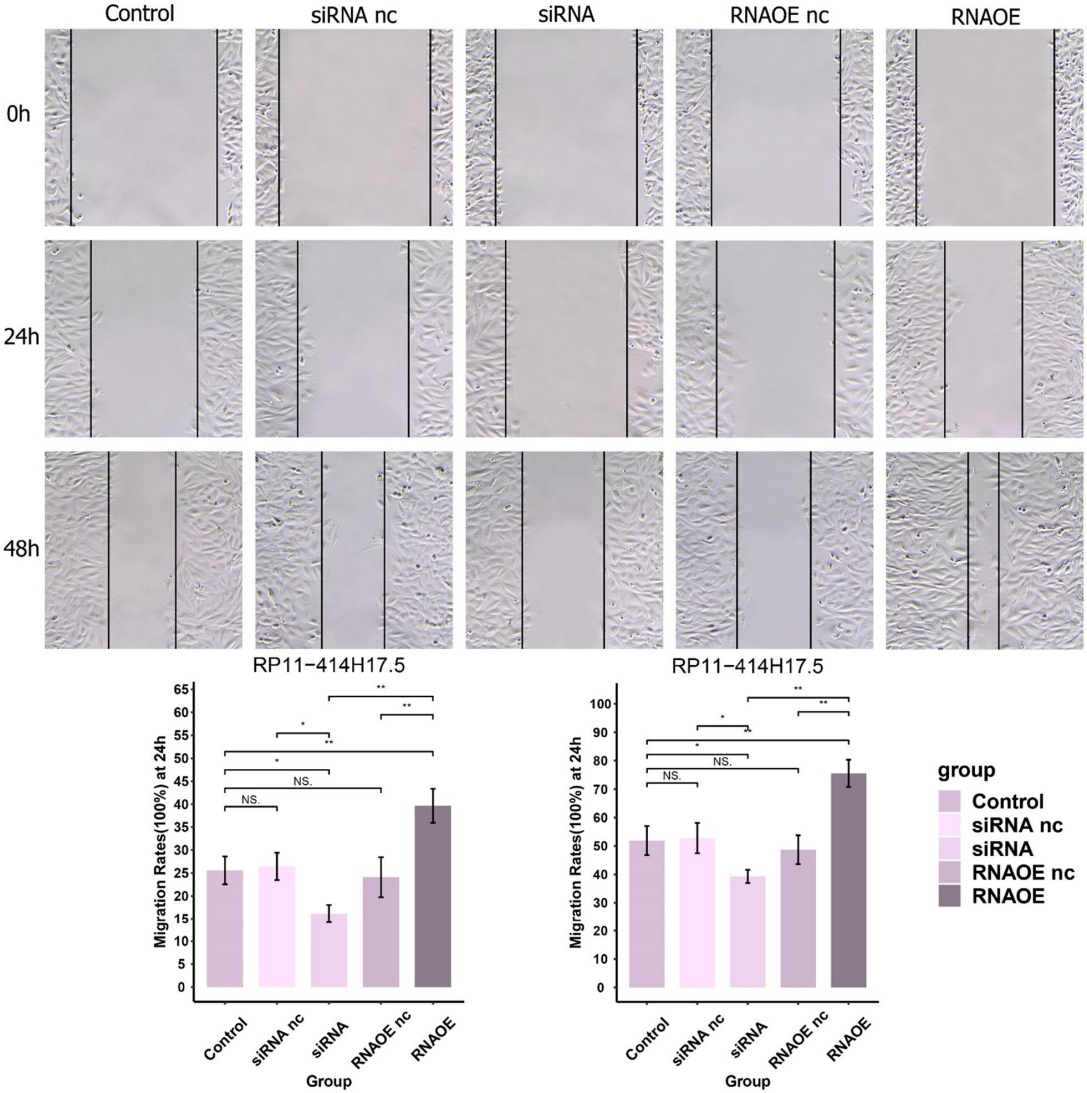
Knockdown of RP11-414H17.5 significantly inhibited the migration ability of Saos-2 cells, and overexpression of RP11-414H17.5 significantly promoted the migration ability of Saos-2 cells

For further validation of the effect of RP11-414H17.5 on osteosarcoma progression, we knocked down and overexpressed RP11-414H17.5 and transfected Saos-2 cells for subsequent experiments. The results indicated that RP11-414H17.5 was successfully knocked down in the siRNA group (Fig. 15A), while that in the RNAOE group was successfully overexpressed (Fig. 15B) (p < 0.05). Figure 16 shows the results of flow cytometry assays on Saos-2 cells with different transfection types, confirming the inhibitory effect of RP11-414H17.5 on osteosarcoma cell apoptosis. Wound-healing assays indicated that RP11-414H17.5 promoted the migration of osteosarcoma cells at both 24 and 48 h (Fig. 17).

## Discussion

Osteosarcoma is a rare malignant tumor of the bone, which typically occurs at two peak periods, adolescence and old age [31]. Despite standard treatment options for osteosarcoma including surgical resection and perioperative chemotherapy, their recurrence rate and survival of metastatic osteosarcoma remain dismal [32, 33]. Due to the lack of reliable prognostic markers, screening for osteosarcoma remains focused on high-risk groups [34, 35]. Osteosarcoma requires X-ray and biopsy samples for definitive diagnosis and CT examination to determine the presence of distant metastasis [36, 37], which imposes a heavy financial burden on both society and individuals. Circadian rhythms involve the regulation of complex physiological mechanisms in humans, including DNA repair, cellular metabolism, apoptosis, stemness, and other biological processes are affected [38, 39]. Numerous studies have shown that circadian rhythms have powerful effects on tumor progression [40, 41], affecting the tumor microenvironment and immune regulation [42–44], and the oncogenic effects of circadian rhythm disruption have been widely recognized [45, 46]. LncRNAs have important regulatory functions in cell differentiation, migration, and apoptosis [13, 47], and have been widely studied as therapeutic targets for various tumors in recent years [48–51]. However, fewer researches have been published on the impact of circadian rhythms on osteosarcoma. Kong et al. [52] found that the circadian rhythm-related gene ARNTL was a protective factor for the prognosis of osteosarcoma patients, and that ARNTL was positively correlated with DNA repair genes and was involved in the biological process of DNA damage repair in osteosarcoma patients. Zhou et al. [53] found that Cry1 was involved in the regulation of the clock gene network and facilitated the proliferation and migration in human osteosarcoma cells in an Akt-dependent manner. But none of the above studies elaborated on the role played by lncRNAs in this process. Therefore, we focused on the relationship between circadian rhythm-associated lncRNAs and osteosarcoma progression, and dissected their value in the prognostic management of osteosarcoma.

We searched the Genecard website for the keyword "circadian" and filtered out genes with protein coding function and correlation score > 1. This resulted in 943 circadian rhythm-related genes. Through combining the TARGET and GTEx databases, this study constructed a circadian rhythm-associated osteosarcoma risk model CRPS for the first time, including high-risk lncRNAs RP11-136I14.5, RP11-414H17.5, and a low-risk lncRNA RP11-750H9.5. The correlation node plot showed that RP11-750H9.5 was negatively correlated with the remaining DECRLs. We tested and validated the prognostic predictive ability of CRPS by survival status scatter plot, risk DECRLs expression heatmap, ROC curve, and KM curve. The AUC values of CRPS for predicting prognosis of osteosarcoma were higher than other clinical features in all cohorts, test set, and training set, and all cohorts. Survival was significantly different between risk subgroups, as demonstrated by the KM curves. The risk heat map showed that RP11-750H9.5 expression was higher in low-risk group, whereas RP11-136I14.5 and RP11-414H17.5 expression was higher in high-risk group. We then found that CRPS and metastasis could be considered as independent prognostic factors in osteosarcoma patients through univariate and multivariate COX analyses. KM curves for the different clinical characteristics groups showed significant differences in survival status of all groups except for the metastasis group, suggesting that CRPS is highly applicable to different clinical subgroups. We considered that the possible reason for lacking significant difference in metastasis group might be the small sample size, but the trend was consistent with all groups. The PCA results confirmed that CRPS can well distinguish different risk subgroups. We built a nomogram for predicting osteosarcoma patients’ survival at different time points based on CRPS and all clinical traits, and ROC curve, C-index curve, the calibration curve all argued for the strong predictive ability of this nomogram. Enrichment analyses of DEGs between different risk subgroups show that these genes are primarily enriched in pathways such as primary immunodeficiencies, T cell receptor signaling pathways, Th1 and Th2 cell differentiation, cell adhesion molecules, antigen processing and expression, and Th17 cell differentiation, which suggests that there is a likelihood that circadian rhythm-associated lncRNAs will influence osteosarcoma progression through immune pathways.

In addition, an independent study of risk DECRLs was conducted. Box plots show that all three risk DECRLs were significantly differentially expressed in different risk subgroups. RP11-414H17.5 was a high-risk DECRL with the highest risk coefficient, while RP11-750H9.5 was the only low-risk DECRL, and box plots and ROC curves were plotted for both as a means of exploring their correlation with osteosarcoma metastasis. The results demonstrate that RP11-414H17.5 expression was significantly higher in the metastasis group, with an AUC value of 0.713 for the prediction of osteosarcoma metastasis, while RP11-750H9.5 expression was significantly higher in the non-metastasis group with an AUC value of 0.709 for the prediction of non-metastasis of osteosarcoma. The correlation of RP11-414H17.5 with metastatic-related genes of osteosarcoma was further investigated. The correlation scatter plots indicated that RP11-414H17.5 had positive correlation with genes promoting osteosarcoma metastasis (as for instance ALDOA, EZH2, IRS1, CUL1, EZR, DNMT1, etc.), and negatively correlated with the osteosarcoma metastasis suppressor gene, TNFSF10, suggesting that RP11-414H17.5 is probably a promoter of osteosarcoma metastasis. RP11-414H17.5 was knocked down and overexpressed and osteosarcoma cells were transfected. After successful knockdown and overexpression, it was observed that both migratory ability and apoptosis of Saos-2 cells were significantly influenced. Flow cytometry shows that RP11-414H17.5 inhibited osteosarcoma cell apoptosis, while wound healing assay indicates that RP11-414H17.5 promoted osteosarcoma cell migration, which is in accordance with previous findings. In order to probe the possible biologic processes involved in RP11-414H17.5, enrichment analyses were conducted on DEGs between different expression subgroups of RP11-414H17.5. GO enrichment analyses showed significant enrichment of these genes in the immunoglobulin production, leukocyte-mediated immunity, complement activation, B cell receptor signaling pathway, B cell activation and other immune-associated pathways, as well as significantly enriched in KEGG pathways including bladder cancer and renal cell carcinoma, which suggests that RP11-414H17.5 may contribute to the progression of various tumors by affecting the immune status.

Many scholars have previously explored the effects of circadian rhythm-associated genes on different tumors. Brooks et al. [54] found that circadian rhythm-related lncRNA ADIRF-AS1 drove clear cell renal carcinoma progression through targeting PBAF; Zhang et al. [55] constructed a model of circadian rhythm-associated lncRNAs that stably predicted the prognosis of hepatocellular carcinoma with a significant impact to the immune infiltration status of the tumor; Liu et al. [56] constructed a circadian rhythm-associated gene risk model to predict survivals in patients with melanoma, non-small-cell lung cancer, and glioma, and could differentiate the immune cell infiltration status in these patients. This research is the first to construct a circadian rhythm-associated lncRNAs prognostic prediction model for the precise prediction of prognosis in osteosarcoma patients. As a complement to preoperative chemotherapy and surgical resection, postoperative immunotherapy for osteosarcoma is playing a critical part in improving patients' five-year survival [57–59]. Circadian rhythm-related genes have also been previously found to regulate the immune infiltration status of tumors [18, 60–62]. In order to explore how CRPS affects the osteosarcoma immune microenvironment, the immune infiltration status and immune checkpoints between different risk subgroups were analyzed, providing equally encouraging results. The immunological scores of CD8 + T cells, B cells, neutrophils, macrophages, T helper cells, and T cell co-inhibition in the high-risk group were found to be significantly lower, together with significantly lower levels of CD44, CD40, PDCD1LG2, LAG3, HAVCR2, and LGALS9 immune checkpoints expression. Previous studies have also found that CD44, CD40, PDCD1LG2, LAG3, and HAVCR2 can be immunotherapeutic targets for osteosarcoma [63–67]. The low-risk group has higher Stromalscore, Immunescore and ESTIMATEscore as well as better immune infiltration. Meanwhile, we found that RP11-750H9.5, as a low-risk DECRL, positively and significantly correlated to the immunological infiltration score, while both RP11-136I14.5 and RP11-414H17.5, as high-risk DECRLs, were negatively associated with the immune infiltration score. These findings are all consistent with our previous enrichment analysis, suggesting that CRPS probably influences osteosarcoma progression through immune-related pathways including the activation of T cells and B cells. Chi et al. [68] built a prognostic prediction model of circadian rhythm-related genes for accurately predicting the prognosis and immunological status of head and neck squamous cell carcinoma patients; Wang et al. [60] found that biological clock gene expression dysregulation promotes glioma progress through impacting the tumor immune landscape; Sun et al. [69] constructed a circadian rhythm-associated prognostic prediction model for hepatocellular carcinoma, which guided immunotherapy and chemotherapy strategies for hepatocellular carcinoma patients. Our study identified circadian rhythm-associated lncRNAs significantly affecting immune infiltration and immune checkpoints in osteosarcoma for the first time, confirming that CRPS predicts the osteosarcoma immune infiltration and guides immunotherapy for patients.

Analyses were subsequently performed on the susceptibility of different risk subgroups to chemotherapeutic agents, demonstrating that the low-risk group patients had greater susceptibility to HG-6-64-1, WH-4-023, and WZ-1-84, while the high-risk group patients exhibited more sensitivity toward SB52334, indicating that CRPS can guide the selection of personalized chemotherapeutic agents for osteosarcoma patients. WH-4-023 has been previously discovered to be valuable for diseases including osteosarcoma and acute lymphoblastic leukemia [70–72].

It has been gradually proven that circadian rhythm disruption affects the progression of a variety of tumors, and this study investigated the factors associated with osteosarcoma progression from a novel perspective, exploring the relationship between circadian rhythm, osteosarcoma, and lncRNA. However, due to the limited sample size of the osteosarcoma database, we were unable to validate it against external datasets. We attempted dataset searches of the GEO database for osteosarcoma samples, but none of these found datasets containing expression information of the risk lncRNAs we screened. We also encourage more scholars to share their datasets for more in-depth studies of these risk lncRNAs. In addition, we were unable to experimentally validate the other two risk lncRNAs due to the condition limitation, so we also hope that more scholars will pay attention to these two lncRNAs in the future and explore their functions and pathogenic mechanisms in depth. In conclusion, we built a robust risk signature that could be used for immune infiltration prediction, prognostic prediction, and individualized guidance of immune targets and chemotherapy in osteosarcoma, and we also found the promoting effect of RP11-414H17.5 on osteosarcoma progression, which is instructive for surveillance and therapy of osteosarcoma.

## Conclusion

This work elucidated the effects of circadian rhythm-associated lncRNAs on osteosarcoma progression for the first time. It found RP11-414H17.5 to be able to elevate osteosarcoma cell malignancy and serve as a therapeutic target for osteosarcoma. This research provides an important reference for the clinical management of osteosarcoma.
